# Active Ingredients of Epimedii Folium and Ligustri Lucidi Fructus Balanced GR/HSP90 to Improve the Sensitivity of Asthmatic Rats to Budesonide

**DOI:** 10.1155/2017/7961231

**Published:** 2017-05-11

**Authors:** Xiufeng Tang, Xiaoxi Li, Honglei Nian, Yan Yang, Yuheng Chen, Xiujuan Wang, Liping Xu, Xinwei Yang, Renhui Liu

**Affiliations:** Beijing Key Lab of TCM Collateral Disease Theory Research, School of Traditional Chinese Medicine, Capital Medical University, No. 10 Xitoutiao, Youanmenwai, Fengtai District, Beijing 100069, China

## Abstract

This study aimed to investigate the possible molecular mechanisms of active ingredients of Epimedii Folium (EF) and Ligustri Lucidi Fructus (LLF) combined with Budesonide (Bun) in asthmatic rats. Rats were divided into 5 groups, including normal group, asthma model group, Bun group, group of active ingredients of EL and LLF (EL), and group of coadministration of Bun with EL (Bun&EL). The asthmatic model was prepared by ovalbumin sensitizing and challenging. Lymphocyte apoptosis, GR protein and binding, and the protein and mRNA of GR*α*, GR*β*, and HSP90 were tested. The results showed that Bun&EL ① markedly increased lymphocyte apoptosis, GR and HSP90 protein, and GR binding in BALF and ② enhanced the expressions of GR*α* and HSP90 and the ratio of GR*α* to GR*β* or to HSP90 both in protein and in mRNA levels in lung, ③ while decrease occurred in GR*β* mRNA and the mRNA ratio of GR*β* to HSP90 compared with asthma or Bun group. Moreover, there was a significant correlation between GR*α* and GR*β* in protein level, or between GR*α* and HSP90 both in protein and in mRNA levels. EL may effectively enhance the sensitivity of asthmatic rats to Bun via balancing GR/HSP90. And these findings will be beneficial for the treatment of asthma in the future.

## 1. Introduction

Asthma is one of the most serious worldwide public health problems that affects all age groups [1] and is characterized by chronic airway inflammation [2]. Glucocorticoids (GCs), which have powerful anti-inflammatory effects, are thought to be the first choice for the prevention and treatment of asthma, especially the application of inhaled GCs, playing anti-inflammatory effects and reducing the systemic side effects of GCs in a certain extent [3, 4]. But some asthma patients with treatment of inhaled GCs at conventional doses may experience no curative effects. When inhaled GCs are administered at high doses, or in long-term using, a series of side effects will be produced, such as growth stunting in children, hormone dependence, hormone resistance, and hypothalamic-pituitary-adrenal (HPA) axis dysfunction [5–7]. In particular, in the face of the occurrence of GC resistance, the current treatment would cost very much due to the lack of effective alternative therapy [8].

How to increase the sensitivity of GC actions in the airway of asthma patients and reduce its adverse reactions has a great significance for the treatment of asthma. GCs mediate their effects via binding to glucocorticoid receptor (GR). GR depends on the heat shock protein 90 (HSP90) molecular chaperone for in vivo function. HSP90 is required for GR to bind ligand and become active and can promote GR-GC binding capacity [9, 10]. So the expression of GR and HSP90 is closely related to the pharmacological effects of GCs.

The traditional Chinese formula, combined Epimedii Folium (EF) and Ligustri Lucidi Fructus (LLF), following the TCM theories and clinical experience, has been used to treat asthmatic patients in China for three decades [11]. Preclinical studies reported that combined EF and LLF (EF&LLF, ET) was able to decrease airway inflammation and airway hyperresponsiveness, adjust the disequilibrium of Th1/Th2, and protect HPA axis in asthmatic rats [12]. In our previous researches, we demonstrated that the decoction of combined EF and LLF (EL) in combination with dexamethasone (Dex) had a better anti-inflammatory effect on the ovalbumin- (OVA-) induced asthmatic rats and could prevent the inhibition of HPA axis and loss of bone mass compared with only using Dex [13, 14]. The effects of EL combined with inhaled GCs on asthma are not clear.

The active ingredients of EL are mainly flavonoids and iridoid, including icariin and oleanolic acid. Icariin possesses extensive therapeutic effects such as protecting neurons from injury, promoting growth of neuronal synapse, regulating of nuclear receptors, and improving sexual dysfunction and bone morphogenesis, as well as anti-inflammation, antitumor, and antidepression functions [15]. Oleanolic acid is able to protect against chemically induced liver injury and has the effects of anti-inflammatory and antihyperlipidemic properties, antitumor-promotion, inhibition of toxicant activation, and the enhancement of the body defense systems [16]. Based on the above understanding about GR/HSP90 in the efficacy of GC, we supposed that the active ingredients of EL could improve GR/HSP90 function to achieve the increased sensitivity of asthma rats to GCs. Therefore, we investigated the effects of coadministration of active ingredients of EL and Budesonide (Bun) on GR, GR isoforms and HSP90, and evaluated the correlation between GR and HSP90 in the lung tissue of asthma rats.

## 2. Material and Methods

### 2.1. Preparation of Active Ingredients

Epimedii Folium (the dried leaf of* Epimediium brevicornu *Maxim) and Ligustri Lucidi Fructus (the dried mature seed of* Ligustrum lucidum *Ait.) were purchased from Beijing* Tongrentang* Pharmaceutical Co. Ltd., China. The two herbs were seriously authenticated and standardized through their marker compounds according to the Chinese Pharmacopoeia (2015) and stored in a dry and sealed container at 4°C to prevent herbs from moisture and moth.

Preparation of active ingredients of EF and LLF, including flavonoids and iridoids, was performed according to the methods described before [17]. And the extraction process of the combined active ingredients has been protected by the Chinese patent (20140037992.5). EF (1000 g) was extracted three times with 90% ethanol (10,000 mL) for 3 h, 2 h, and 2 h at 70°C in a reflux apparatus. The extracts were mixed, filtered, and concentrated under reduced pressure, until recovery to no alcohol precipitation. Then, with petroleum ether extraction three times to remove chlorophyll, the extracts were washed with D-101 macroporous resin to colorless state, eluted with 90% ethanol, and steamed to yield a dark yellow powder. The yield of EF extract was 2.5%. Based on the phytochemical test (*Pharmacopoeia of the People's Republic of China*, 2010 Edition), the content of EF extract was 80% calculated by icariin. LLF (1000 g) was extracted three times with 75% ethanol (10,000 mL) for 3 h, 2 h, and 2 h at 70°C in a reflux apparatus. The extracts were mixed, filtered, and concentrated under reduced pressure, until recovery to no alcohol precipitation. Using AB-8 macroporous resin, the extracts were washed with distilled water and then with 75% ethanol after the water solution was discarded. The steamed extracts are total iridoid of LLF. The yield of Ligustri Lucidi Fructus extracts was 5%. Based on the phytochemical test (*Pharmacopoeia of the People's Republic of China*, 2010 Edition), the content of LLF extracts was more than 80% calculated by oleanolic acid and rutin. The combination of active ingredients of EF and LLF (EL) was mixed at a ratio of 2 to 3, equivalent to the raw herbs ratio of 4 to 3 according to clinical practice. The combined active fractions were dissolved in distilled water at appropriate concentrations.

### 2.2. Animals

40 male Sprague-Dawley rats, weighing 120 to 130 g with the average age of four or five weeks, were purchased from Vital River Laboratory Animal Technology Co. Ltd. (Beijing, China). The experiment complied with the Animal Management Rule of the Ministry of Public Health, China, and the experimental protocol was approved by the Animal Care Committee of Capital Medical University, Beijing, China. All the animals were cared for in the Experimental Animal Center of Capital Medical University. During the whole experiment, the animals were housed in stainless cages (three rats per cage) at conventional controlled conditions (temperature of 23 ± 2°C, relative humidity of 50 ± 10%, and 12-hour light-dark cycle). They were allowed free access to the standard laboratory food and tap water.

### 2.3. Experimental Protocol

After acclimatization for 7 days, the rats were randomly assigned into 5 groups (*n* = 8 per group): normal control group, asthma model group, Bun group, EL group, and group of coadministration of Bun with EL (Bun&EL).

OVA sensitization and challenge protocols were performed according to the methods of Yang et al. [18] with certain modifications as described below. All the rats with the exception of those in the normal control group were actively sensitized with an intraperitoneal (i.p.) injection and subcutaneous injection of 1 mg OVA (Grade II, Sigma-Aldrich, St. Louis, MO, USA) and 100 *μ*g aluminum hydroxide in 1 mL sterile physiological saline. After seven days, the same procedure was repeated. After another seven days, the OVA-sensitized rats were exposed to 1% aerosolized OVA (1 g OVA in 100 mL sterile physiological saline in an ultrasonic nebulizer) for 30 min once a day. Three weeks later, the OVA-sensitized rats were challenged twice a week and treated once a day. Rats in the Bun and Bun&EL groups were exposed to Bun suspension (AstraZeneca Pty Ltd., New South Wales, Australia) for inhalation (1 mg Bun suspension in 50 mL sterile physiological saline in an ultrasonic nebulizer) for 30 min. Rats in the EL and Bun&EL groups were administered with the active ingredients of EL at the dose of 100 mg/kg body weight. At the same time, the rats in the normal control group and asthma group were given the same volume of distilled water.

On the 64th day, all the rats were anesthetized with 25% ethyl carbamate (4 mL/kg, i.p.) 24 h after the last OVA exposure. Blood samples were collected by abdominal aortic puncture. Lungs were lavaged 3 times with 3 mL, 3 mL, and 4 mL ice-cold saline using a tracheal cannula and a 5 mL polyethylene syringe. The cell-debris pellets of bronchoalveolar lavage fluid (BALF) samples were collected after centrifugation (500 rpm, 5 min, and 4°C). Lymphocytes were separated from blood and BALF samples with assay of rat lymphocyte separation solution (Tianjin Hao Yang Biological Manufacture Co., Ltd., Tianjin, China) and then resuspended with 1 mL of 4% paraformaldehyde-phosphate buffer solution.

### 2.4. TUNEL Assay for Lymphocyte Apoptosis

Lymphocytes apoptosis in blood and BALF was determined using the TUNEL assay (MBL, Inc., Nagoya, Japan) for flow cytometry (FCM) according to manufacturer's instruction. A FACSCalibur flow cytometer (BD, Lake Franklin, NJ, USA) was used to determine lymphocyte apoptosis level through mean fluorescence intensity (MFI).

### 2.5. GR Protein in BALF Lymphocyte by FCM

The samples of 300 *μ*L lymphocyte resuspension from BALF were washed with PBS twice and refixed in 100 *μ*L of 4% buffered paraformaldehyde for 30 min at 4°C. Nonspecific binding site was blocked with 5% normal goat serum containing permeabilisation buffer for 30 min. The lymphocytes were then incubated with 100 *μ*L permeabilisation buffer containing 50 *μ*L anti-GR mAb (Abcam, Cambridge, UK; 1 : 100) for 60 min, washed twice with permeabilisation buffer, and then incubated with 100 *μ*L permeabilisation buffer containing 50 *μ*L of goat anti-mouse IgG- FITC (Caltag, Burlingame, CA, USA; 1 : 100) for 60 minutes. After extensive washing with permeabilisation buffer to remove unbound secondary antibodies, the lymphocytes were resuspended in 500 *μ*L fixation buffer. Lymphocyte samples were run on a FACSCalibur FCM. The relative quantity of GR protein (mean fluorescence) was expressed as MFI.

### 2.6. GR Binding in BALF Lymphocyte by FCM

The samples of 300 *μ*L lymphocyte resuspension from BALF were washed with PBS and resuspended in 100 *μ*L PBS containing 2 × 10^-5 ^M PE-Dex (Molecular Probe, Invitrogen, Carlsbad, CA, USA) for 60 min at 37°C in the dark with gentle mixing every 10 min. As controls, another tube was prepared adding a 500-fold excess amount of unlabelled Dex (Sigma, St. Louis, MO, USA) 10 min before PE-Dex. Finally, the lymphocytes were washed twice and resuspended in 300 *μ*L fixation buffer. Lymphocyte samples were run on a FACSCalibur FCM. The relative quantity of GR binding (mean fluorescence) was expressed as MFI.

### 2.7. Protein of GR*α*, GR*β*, and HSP90 in Lung by Immunohistochemistry (IHC)

GR*α*, GR*β*, and HSP90 were identified in paraffin-embedded sections of the lung tissue by IHC staining with anti-GR*α* (Abcam, Cambridge, UK), GR*β* (Biorbyt, Cambridge, UK), or HSP90 antibody (Abcam, Cambridge, UK) overnight at 4°C at a concentration of 1 : 100 (GR*α* and HSP90) or 1 : 50 (GR*β*) followed by standard biotin-streptavidin-peroxidase immunostaining using a streptavidin-peroxidase kit (Zhongshan Goldenbridge Biotechnology, Beijing, China) following the instructions provided by the manufacturer. Staining was completed by incubation with diaminobenzidine chromogen solution at room temperature. All measurements were performed with the Nikon ECLIPSE 80i biomicroscope and NIS-Elements BR 3.2 image analysis system (Nikon, Japanese). Three random images within a lung sample were taken and further analyzed by using zoomed-in field at 400x magnification. We measured the integral optical density (IOD) and the positive area of GR*α*, GR*β*, and HSP90-positive cells under each examined field and calculated the average number as the final result of this sample.

### 2.8. HSP90 Protein in BALF by Immunocytochemistry (ICC)

To further confirm HSP90 expression in airway, we select ICC analysis to detect HSP90 protein in BALF cell residue. After fixation by cold acetone for 10 min, BALF cytospins were incubated by 3% hydrogen peroxide for 10 min. After being blocked with normal goat serum for 20 min, the samples were incubated with anti-HSP90 antibody (Abcam, Cambridge, UK) at a concentration of 1 : 50 for 1 h at 37°C. Polink-2 Plus Polymer HRP Detection Systems were used and then developed with diaminobenzidine (DAB) kit (Zhongshan Golden Bridge Biotechnology Co., Ltd., Beijing, China). All measurements were performed with the Nikon ECLIPSE 80i biomicroscope and NIS-Elements BR 3.2 image analysis system (Nikon, Japanese).

### 2.9. Protein of GR*α*, GR*β*, and HSP90 by Western Blotting (WB) Analysis

The total protein was extracted from 50 mg lung tissue using RIPA lysis buffer. Concentration of the protein was quantified by quantitative bromochloroacetate (BCA) protein kit (Beijing Biosynthesis Biotechnology Co., Ltd., Beijing, China). The protein was mixed with loading buffer and boiled at 95°C for 5 min to make it denatured. Equal amounts of protein (40 *μ*g per lane) were separated by 10% sodium dodecyl sulfatepolyacrylamide gel electrophoresis (SDS-PAGE) and transferred into 0.45 *μ*m polyvinylidene fluoride (PVDF) membranes. After blocking with 5% nonfat-dried milk at room temperature for 2 h, membranes were incubated with primary antibodies: rabbit polyclonal to HSP90 antibody (Abcam, Cambridge, UK; 1 : 5000), rabbit polyclonal to GR*α* antibody (Abcam, Cambridge, UK; 1 : 2000), rabbit polyclonal to GR*β* antibody (Biorbyt, Cambridge, UK; 1 : 2000), and mouse monoclonal to *β*-actin antibody (Zhongshan Golden Bridge Biotechnology Co., Ltd., Beijing, China; 1 : 2000) overnight at 4°C. Membranes were detected with horseradish peroxidase-labeled goat anti-rabbit IgG (Zhongshan Golden Bridge Biotechnology Co., Ltd., Beijing, China; 1 : 20000) or goat anti-mouse IgG (Zhongshan Golden Bridge Biotechnology Co., Ltd., Beijing, China; 1 : 40000) as the secondary antibody for 1 h. The protein band was visualized by an electrochemiluminescent (ECL) reagent and exposed to X-film. *β*-Actin was used for normalization. The sumdensity of each protein band was measured by ImageJ software (National Institutes of Health, USA).

### 2.10. mRNA of GR, GR*α*, GR*β*, and HSP90 by Quantitative Real-Time PCR (qPCR)

Total RNA was isolated from the lung tissue using TRIzol reagent (Life Technologies, Carlsbad, CA, USA) according to the manufacturer's recommendations. Following purification with an RNeasy kit (Qiagen, Valencia, CA, USA), M-MLV reverse transcription (Promega, Madison, USA) was used to synthesize complementary DNA (cDNA). The qPCR amplification was performed using the SYBR-green detection of PCR products in real time with an ABI-7500 Sequence Detection System (Applied Biosystems, Foster City, CA, USA). The primers used in the qPCR analysis are presented in Table 1. The PCR program was performed for 40 cycles with each cycle consisting of 5 min of predenaturation at 94°C, 15 s of denaturation at 94°C, and 30 s of annealing at 60°C. Gene expression was quantified by means of the comparative Ct method (^ΔΔ^Ct) and the relative quantification (RQ) was calculated as 2^−ΔΔCt^. Relative mRNA levels of GR, GR*α*, GR*β*, and HSP90 were examined and normalized to 18S mRNA expression in each sample. The melting curves for each PCR were generated to ensure the purity of the amplification product. A no-template negative control was included in each experiment.

### 2.11. Statistical Analysis

Results of all measurements were presented as means ± standard deviation (SD). The data analysis was performed using the SPSS 13.0 (SPSS Inc., Chicago, USA). All of the data were tested for normality using the Kolmogorov-Smirnov test and passed. A one-way analysis of variance (ANOVA) was performed to determine whether there were statistically significant differences (*P* < 0.05) among the experimental groups. The least significant difference (LSD) test when the variances are equal or Tamhane's T2 test when the variances are not equal was used for comparisons between individual groups and to determine which means differed statistically significantly (*P* < 0.05).* Pearson* correlation analysis was used for detecting the correlation of data.

## 3. Results

### 3.1. Effects of Coadministration of Bun and EL on Lymphocyte Apoptosis

Both anti-inflammatory effect and side effect of GCs were related to inducing apoptosis in autologous mixed lymphocytes [19, 20]. So the level of lymphocyte apoptosis could indicate the sensitivity of Bun to asthmatic inflammation. In this study, we used TUNEL assay to detect lymphocyte apoptosis in BALF and peripheral blood by FCM.

**Figure 1:**
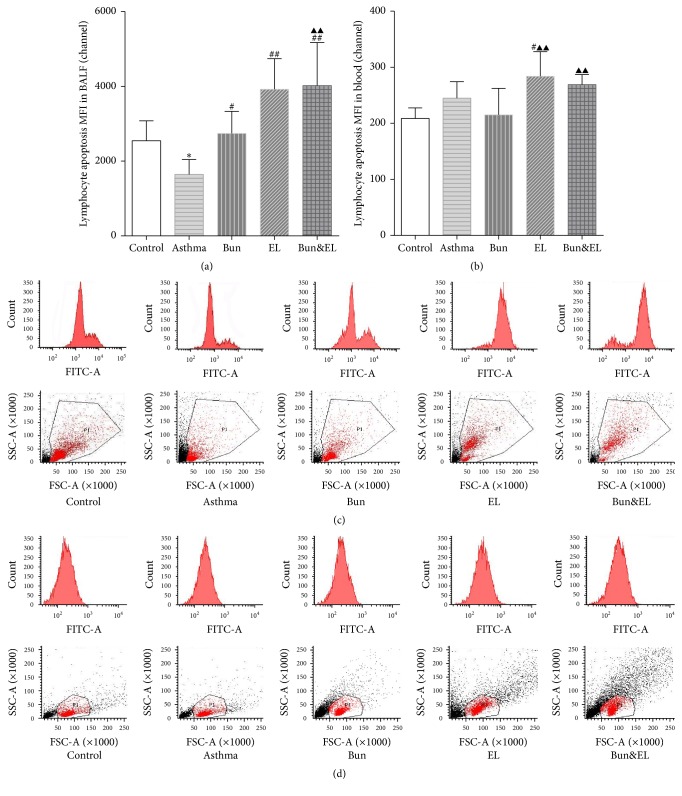
Effects of Bun and EL on lymphocyte apoptosis in blood and BALF. The levels of lymphocyte apoptosis in BALF and peripheral blood were detected by FCM with TUNEL assay in control, asthma, Bun, EL, and Bun&EL groups. (a) Summarized data of MFI of lymphocyte apoptosis in BALF. (b) Summarized data of MFI of lymphocyte apoptosis in peripheral blood. Representative FCM photographs of lymphocyte apoptosis in BALF (c) and peripheral blood (d), including the single parameter histogram and the dot plots of SSC versus FSC, were viewed in control, asthma, Bun, EL, and Bun&EL groups. Values are expressed as mean ± SD. *n* = 7 in control, asthma, Bun, EL, and Bun&EL group, respectively. ^*∗*^*P* < 0.05 versus control group; ^#^*P* < 0.05 and ^##^*P* < 0.01 versus asthma group; ^▲▲^*P* < 0.01 versus Bun group.

The level of lymphocyte apoptosis in BALF, which was significantly lower in the asthma model group than in the control group, was shown in Figure 1(a) (*P* = 0.032). Treatment with Bun, EL, and Bun&EL significantly increased BALF lymphocyte apoptosis compared with those of the asthma group (*P* = 0.010, *P* = 0.000, and *P* = 0.000). In addition, significant difference in lymphocyte apoptosis in BALF was found between the Bun group and the Bun&EL group (*P* = 0.006). These results indicated the coadministration of Bun and EL could prevent the inhibition of BALF lymphocyte apoptosis in asthmatic rats and led to a synergistic upregulation.

### 3.2. Effects of Bun and EL on GR

Lymphocytes and GRs are the best candidates for researching the cellular and molecular targets of GCs. The sensitivity of GRs in lymphocytes may account, in part, for the immunosuppressive efficacy of GCs [21]. Abnormalities of GR seem to be related to steroid resistance, that is, the lower sensitivity of anti-inflammatory effects of GCs [22]. In this study, FCM was used to assess GR expression and binding capacity in BALF lymphocyte, and qPCR was used to detect GR mRNA level in lung tissue.

**Figure 2:**
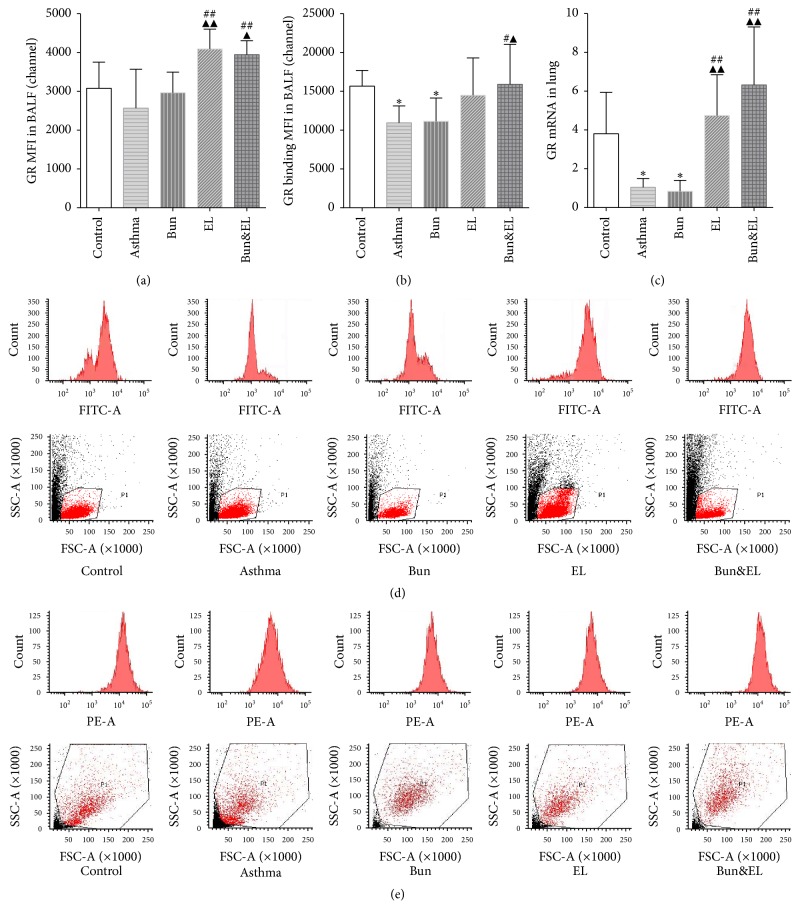
Effects of Bun and EL on GR. GR protein and binding capacity in BALF were detected by FCM in control, asthma, Bun, EL, and Bun&EL groups. (a) Summarized data of MFI of GR protein in BALF. (b) Summarized data of MFI of GR binding capacity in BALF. (c) The mRNA expression of GR was measured by qPCR analysis with 18S as an internal control. Representative FCM photographs of GR protein (d) or GR binding capacity (e) in BALF, including the single parameter histogram and the dot plots of SSC versus FSC, were viewed in control, asthma, Bun, EL, and Bun&EL groups. Values are expressed as mean ± SD. *n* = 6 in control, asthma, Bun, EL, and Bun&EL group, respectively. *P* < 0.05 versus control group; ^#^*P* < 0.05 and ^##^*P* < 0.01 versus asthma group; ^▲^*P* < 0.05 and ^▲▲^*P* < 0.01 versus Bun group. ^*∗*^*P* < 0.05 versus control group.

Figure 2(a) showed that there were no significant differences in GR protein expression of BALF lymphocyte between the control and asthma groups (*P* = 0.1881). GR protein in BALF lymphocyte greatly increased following administration of EL or Bun&EL versus asthma or Bun group (versus asthma group: *P* = 0.000 and *P* = 0.001; versus Bun group: *P* = 0.006 and *P* = 0.015).

As shown in Figure 2(b), GR binding in BALF lymphocyte was significantly decreased after modeling (*P* = 0.036), which signified the inhibition of binding capacity between GC and GR. Following repeated Bun administration, GR binding in BALF lymphocyte was still in a state of suppression in contrast with control group (*P* = 0.043). However, significant increase in GR binding of BALF lymphocyte was noticed in the Bun&EL group compared with the asthma or Bun group (*P* = 0.028 or *P* = 0.033). It was induced that coadministration of Bun and EL could markedly raise the binding capacity between GC and GR.

As shown in Figure 2(c), GR mRNA in lung tissue was dramatically decreased in asthma rats versus the control group (*P* = 0.020). Following repeated Bun administration, GR mRNA was still in a state of suppression in contrast with the control group (*P* = 0.013). However, GR mRNA expression was significantly increased in the EL and Bun&EL groups compared with the asthma or Bun group (versus asthma group: *P* = 0.003 and *P* = 0.002; versus Bun group: *P* = 0.002 and *P* = 0.000). We inferred that coadministration of Bun and EL could markedly raise GR mRNA expression in lung.

### 3.3. Effects of Bun and EL on GR*α*

GCs act through the intracellular GR signals into changes in gene expression [23]. One single GR gene gives rise to two main GR products, namely, GR*α* and GR*β*, which are subject to translational and posttranslational modifications. GR*α* is expressed in virtually all human cells and tissues, including respiratory epithelial cells, and is downregulated by GC at least in vitro. GR*α* is ubiquitously expressed and is responsible for the induction and repression of target genes [24]. GR*α* expression was positively correlated with the anti-inflammatory effect of GC.

**Figure 3:**
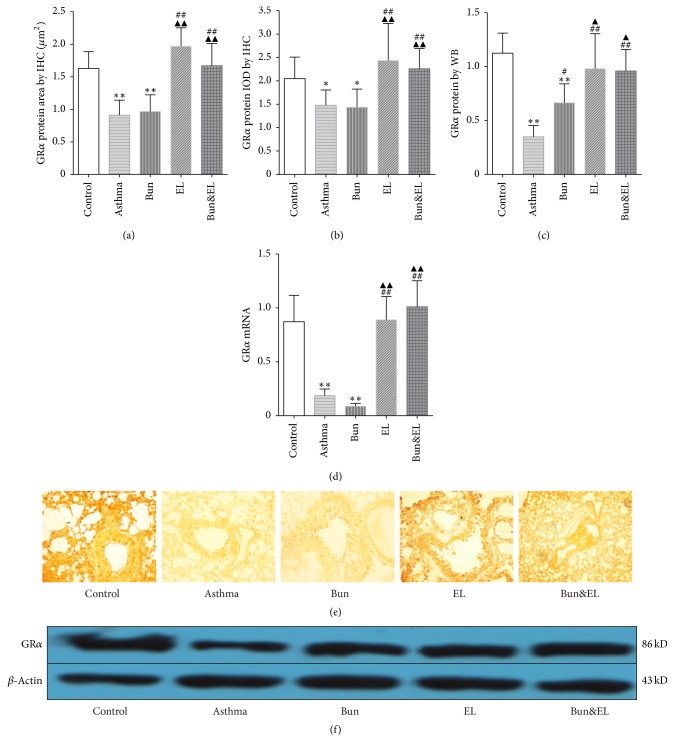
Effects of Bun and EL on GR*α*. GR*α* protein and mRNA expressions in lung tissues were detected by IHC, WB, and qPCR. (a) Summarized data of positive area of GR*α* protein expression in lung tissues measured by IHC. (b) Summarized data of IOD of GR*α* protein expression in lung tissues measured by IHC. (c) Summarized data of sumdensity of GR*α* protein expression in lung tissues measured by WB, normalized to *β*-actin. (d) The mRNA expression of GR*α* was measured by qPCR analysis with 18S as an internal control. (e) Representative IHC photographs of GR*α* were viewed at a magnification of 400x in control, asthma, Bun, EL, and Bun&EL groups. (f) Representative WB photographs of GR*α* were viewed, and *β*-actin was used for normalization in control, asthma, Bun, EL, and Bun&EL groups. Values are expressed as mean ± SD. *n* = 7 (a, b), *n* = 5 (c), or *n* = 6 (d). ^*∗*^*P* < 0.05 and ^*∗∗*^*P* < 0.01 versus control group; ^##^*P* < 0.01 versus asthma group; ^▲^*P* < 0.05 and ^▲▲^*P* < 0.01 versus Bun group. ^#^*P* < 0.05.

As shown in Figures 3(a) and 3(b), positive area and integral optical density (IOD) of GR*α* protein expression detected by IHC were significantly decreased in asthma and Bun groups versus the control group (positive area: both *P* = 0.000; IOD: *P* = 0.045 and *P* = 0.032). However, GR*α* positive area and IOD were remarkably increased in EL and Bun&EL groups compared with the asthma or Bun group (all *P* < 0.01).

GR*α* protein expression was further confirmed by WB analysis (Figure 3(c)). GR*α* protein was downregulated in asthma and Bun groups versus the control group (*P* = 0.000 and *P* = 0.003). There was a significant increase in GR*α* protein in Bun, EL, and Bun&EL groups compared with the asthma group (*P* = 0.027, *P* = 0.000, and *P* = 0.000). The coadministration of Bun and EL significantly increased GR*α* protein expression versus the Bun group (*P* = 0.039).

GR*α* mRNA expression was detected by qPCR analysis (Figure 3(d)). GR*α* mRNA expression was markedly decreased in asthma and Bun groups versus the control group (both *P* = 0.000). Compared with asthma or Bun group, significant increase was noticed in GR*α* mRNA in the EL or Bun&EL group (*P* = 0.000).

### 3.4. Effects of Bun and EL on GR*β*

GR*β* acts as a dominant-negative inhibitor of GR*α*-mediated transactivation and transrepression in certain cell types. Increased expression of GR*β*, which competes with and thus inhibits activated GR*α*, has been suggested that GR*β* could cause steroid resistance [25]. Very low GR*β* mRNA levels have been detected in a number of cells and tissues, which often contradict GR*β* protein data. An association between GC insensitivity and increased GR*β* expression has been reported in asthma and in vitro, certain proinflammatory cytokines upregulate GR*β* expression [26]. In this study, the protein and mRNA expressions of GR*β* in the lung tissue were tested by IHC, WB, and qPCR analysis, and an altered ratio in the expression of GR*α* and GR*β* was calculated.

**Figure 4:**
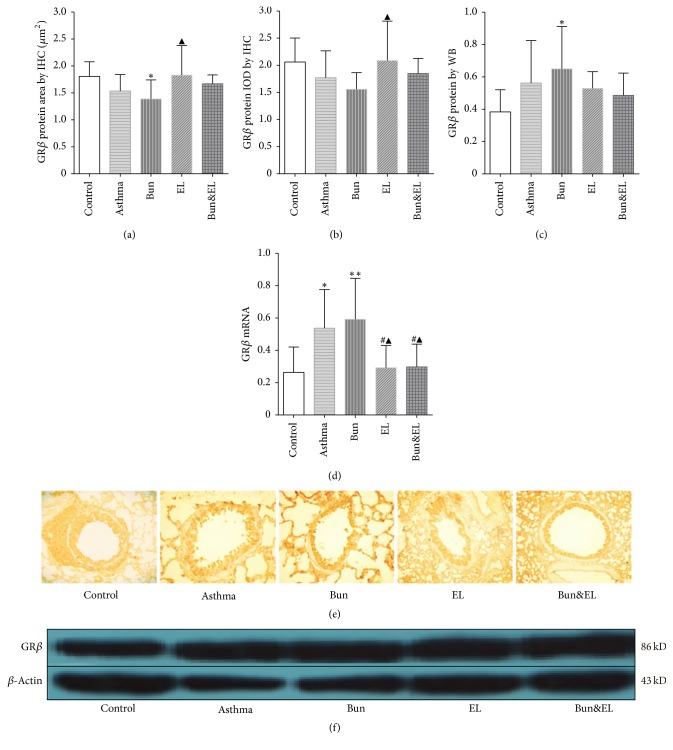
Effects of Bun and EL on GR*β* expression. GR*β* protein and mRNA expressions in lung tissues were detected by IHC, WB, and qPCR. (a) Summarized data of positive area of GR*β* protein expression in lung tissues measured by IHC. (b) Summarized data of IOD of GR*β* protein expression in lung tissues measured by IHC. (c) Summarized data of sumdensity of GR*β* protein expression in lung tissues measured by WB, normalized to *β*-actin. (d) The mRNA expression of GR*β* was measured by qPCR analysis with 18S as an internal control. (e) Representative IHC photographs of GR*β* were viewed at a magnification of 400x in control, asthma, Bun, EL, and Bun&EL groups. (f) Representative WB photographs of GR*β* were viewed, and *β*-actin was used for normalization in control, asthma, Bun, EL, and Bun&EL groups. Values are expressed as mean ± SD. *n* = 7 (a, b), *n* = 5 (c), and *n* = 6 (d). ^*∗*^*P* < 0.05 and ^*∗∗*^*P* < 0.01 versus control group; ^#^*P* < 0.05 versus asthma group; ^▲^*P* < 0.05 versus Bun group.

As shown in Figures 4(a), 4(b), and 4(c), there was no significant change in GR*β* protein expression in lung between the control and asthma groups. GR*β* mRNA (Figure 4(d)) was upregulated in the asthma and Bun groups versus the control group (*P* = 0.021 and *P* = 0.007). However, there was a significant decrease in GR*β* mRNA expressions in the EL and Bun&EL groups compared with the asthma or Bun group (all *P* < 0.05).

**Figure 5:**
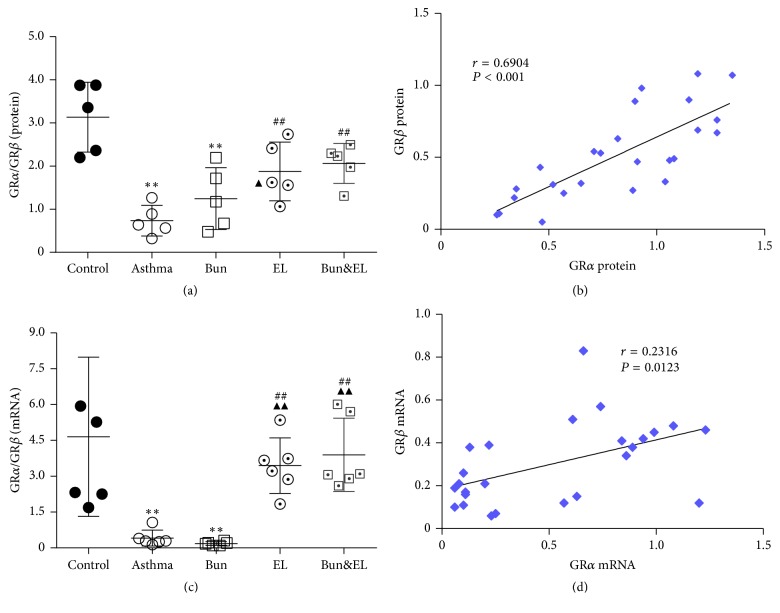
Effects of Bun and EL on correlation between GR*α* and GR*β*. (a) The ratio of GR*α* to GR*β* was calculated in the level of relative protein measured by WB, normalized to *β*-actin. (b) The correlation between GR*α* and GR*β* was analyzed in the level of relative protein measured by WB, normalized to *β*-actin. (c) The ratio of GR*α* to GR*β* was calculated in the level of relative mRNA expression measured by qPCR with 18S as an internal control. (d) The correlation between GR*α* and GR*β* was analyzed in the level of relative mRNA expression measured by qPCR with 18S as an internal control. Values are expressed as mean ± SD. *n* = 5 (a), *n* = 6 (c), *n* = 25 (b), or *n* = 30 (d). ^*∗∗*^*P* < 0.01 versus control group; ^##^*P* < 0.01 versus asthma group; ^▲▲^*P* < 0.01 versus Bun group.

The protein ratio tested by WB (Figure 5(a)) or mRNA ratio (Figure 5(c)) of GR*α* to GR*β* was markedly reduced in the asthma and Bun groups versus the control group (both *P* < 0.01). Compared to the asthma group, GR*α*/GR*β* protein ratios were significantly increased in the EL and Bun&EL groups (both *P* < 0.01). Compared to the asthma or Bun group, GR*α*/GR*β* mRNA ratio was significantly increased in the EL and Bun&EL groups (all *P* < 0.01). In addition, there was a significant correlation between GR*α* and GR*β* in protein level (Figure 5(b)).

### 3.5. Effects of Bun and EL on HSP90

HSP90 associates selectively with unliganded GR, and the proper folding of GR depends on direct interactions with the chaperone, HSP90 [27]. Although an association with HSP90 presumably masks the GR nuclear localization signal sequence, the recent demonstration of the coimport of GR and HSP90 into nuclei has led to the hypothesis that HSP90 facilitates GR interactions with the nuclear transport machinery [28].

**Figure 6:**
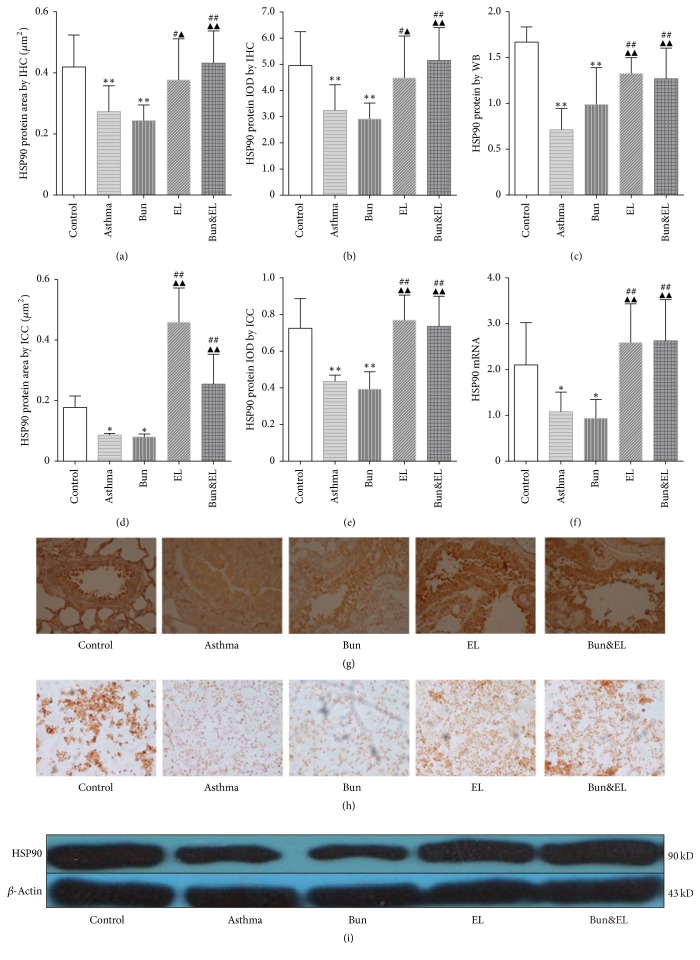
Effects of Bun and EL on HSP90. HSP90 protein and mRNA expressions in lung tissues were detected by IHC, WB, and qPCR. And HSP90 protein expression in BALF was detected by ICC. (a) Summarized data of positive area of HSP90 protein expression in lung tissues measured by IHC. (b) Summarized data of IOD of HSP90 protein expression in lung tissues measured by IHC. (c) Summarized data of sumdensity of HSP90 protein expression in lung tissues measured by WB, normalized to *β*-actin. (d) Summarized data of positive area of HSP90 protein expression in BALF measured by ICC. (e) Summarized data of IOD of HSP90 protein expression in BALF measured by ICC. (f) The mRNA expression of HSP90 was measured by qPCR analysis with 18S as an internal control. (g) Representative IHC photographs of HSP90 were viewed at a magnification of 400x in control, asthma, Bun, EL, and Bun&EL groups. (h) Representative ICC photographs of HSP90 were viewed at a magnification of 400x in control, asthma, Bun, EL, and Bun&EL groups. (i) Representative WB photographs of GR*β* were viewed, and *β*-actin was used for normalization in control, asthma, Bun, EL, and Bun&EL groups. Values are expressed as mean ± SD. *n* = 8 (a, b) and *n* = 6 (c, d, e, f). ^*∗*^*P* < 0.05 and ^*∗∗*^*P* < 0.01 versus control group; ^#^*P* < 0.05 and ^##^*P* < 0.01 versus asthma group; ^▲^*P* < 0.05 and ^▲▲^*P* < 0.01 versus Bun group.

In this report, we examined HSP90 protein expressions in the lung tissue by IHC and WB. As shown in Figures 6(a), 6(b), and 6(c), HSP90 protein was significantly decreased in the asthma and Bun groups versus the control group (all *P* < 0.01). And, compared to the asthma or Bun group, HP90 protein levels were significantly increased in the EL and Bun&EL groups (*P* < 0.05 or *P* < 0.01). To further determine the effects of Bun and EL on HSP90 protein expression in airway, we used ICC method to detect this expression in BALF. As shown in Figures 6(d) and 6(e), HSP90 protein expression in BALF was significantly decreased in the asthma and Bun groups versus the control group (*P* < 0.05 or *P* < 0.01). Compared with the asthma or Bun group, HSP90 protein was significantly increased in the EL and Bun&EL groups (all *P* < 0.01).

HSP90 mRNA in lung was detected by qPCR methods (Figure 6(f)). HSP90 mRNA was markedly decreased in the asthma and Bun groups versus the control group (both *P* < 0.05). Compared with the asthma or Bun group, HSP90 mRNA was significantly increased in the EL and Bun&EL groups (all *P* < 0.01).

### 3.6. Effects of Bun and EL on the Relation between GR Isoforms and HSP90

The ligand binding activity of GR is related to the changes of HSP90 after all. To evaluate the correlation between GR and HSP90 on the asthmatic rat treated with EL combined with Bun, we calculated the ratio of GR isoforms (including GR*α* and GR*β*) to HSP90 and evaluated the association between ligand binding activity of GR and HSP90.

**Figure 7:**
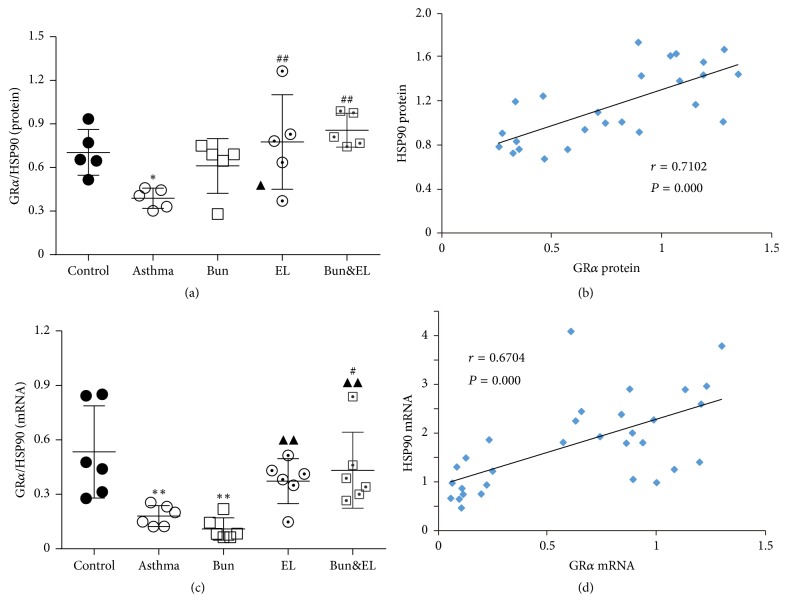
Effects of Bun and EL on correlation between GR*α* and HSP90. (a) The ratio of GR*α* to HSP90 was calculated in the level of relative protein measured by WB, normalized to *β*-actin. (b) The correlation between GR*α* and HSP90 was analyzed in the level of relative protein measured by WB, normalized to *β*-actin. (c) The ratio of GR*α* to HSP90 was calculated in the level of relative mRNA expression measured by qPCR with 18S as an internal control. (d) The correlation between GR*α* and HSP90 was analyzed in the level of relative mRNA expression measured by qPCR with 18S as an internal control. Values are expressed as mean ± SD. *n* = 5 (a), *n* = 6 (c), *n* = 25 (b), and *n* = 30 (d). ^*∗∗*^*P* < 0.01 versus control group. ^#^*P* < 0.05 and ^##^*P* < 0.01 versus asthma group; ^▲^*P* < 0.05 and ^▲▲^*P* < 0.01 versus Bun group. ^*∗*^*P* < 0.05 versus control group.

The ratios of GR*α* to HSP90 in the levels of protein (Figure 7(a)) and mRNA (Figure 7(c)) were markedly reduced in the asthma group versus the control group (*P* < 0.05 and *P* < 0.01). Compared to the asthma group, the ratios were significantly increased in Bun&EL groups (*P* < 0.01 and *P* < 0.05). And compared to the Bun group, GR*α*/HSP90 in the mRNA level was significantly increased in EL or Bun&EL group (both *P* < 0.01). In addition, there was a significant correlation between GR*α* and HSP90 in protein level (*r* = 0.7102, *P* = 0.000; Figure 7(b)) or in mRNA level (*r* = 0.6704, *P* = 0.000; Figure 7(d)).

**Figure 8:**
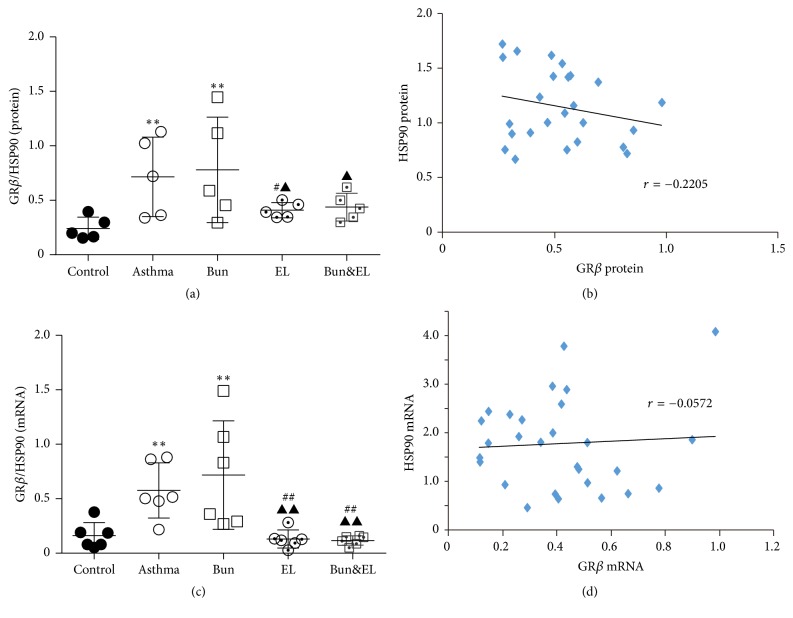
Effects of Bun and EL on correlation between GR*β* and HSP90. (a) The ratio of GR*β* to HSP90 was calculated in the level of relative protein measured by WB, normalized to *β*-actin. (b) The correlation between GR*β* and HSP90 was analyzed in the level of relative protein measured by WB, normalized to *β*-actin. (c) The ratio of GR*β* to HSP90 was calculated in the level of relative mRNA expression measured by qPCR with 18S as an internal control. (d) The correlation between GR*β* and HSP90 was analyzed in the level of relative mRNA expression measured by qPCR with 18S as an internal control. Values are expressed as mean ± SD. *n* = 5 (a), *n* = 6 (c), *n* = 25 (b), and *n* = 30 (d).^*∗∗*^*P* < 0.01 versus control group. ^#^*P* < 0.05 and ^##^*P* < 0.01 versus asthma group; ^▲^*P* < 0.05 and ^▲▲^*P* < 0.01 versus Bun group.

Figures 8(a) and 8(c) showed that the ratios of GR*β* to HSP90 in protein and mRNA levels were markedly higher in the asthma and Bun groups than that in the control group (all *P* < 0.01). In the protein level, GR*β*/HSP90 ratio was lower in the EL and Bun&EL groups versus the Bun group (both *P* < 0.05). In the mRNA level, GR*β*/HSP90 ratio was decreased in the EL and Bun&EL groups compared to the asthma or Bun group (all *P* < 0.01). Figures 8(b) and 8(d) showed that there was no significant correlation between GR*β* and HSP90 in the protein or mRNA level.

## 4. Discussion

Airway inflammation is considered to be the basic pathological change and one of the key pathological mechanisms of repeated attacks of asthma [29, 30]. GCs remain the cornerstone of asthma management because of the most effective anti-inflammatory effect. But it is inevitable that adverse reactions happen with administration of GC with large dose or long protocol. We think that improving the sensitivity of GC to airway inflammation in asthma and reducing the dosage of GC medication will effectively prevent the occurrence of its adverse reactions.

In China, traditional Chinese medicine (TCM) has been widely used to treat asthma with its unique advantages for thousands of years. According to TCM theories, kidney governs qi reception, which means that kidney plays important roles in respiratory function. Because kidney deficiency is the main internal disease causes of asthma, the treatment of asthma in remission stage follows the basic strengthening-kidney rules [31]. ET* (Yinyanghuo)* and LLF* (Nvzhenzi)* were documented as replenishing kidney-yang and kidney-yin herbs, respectively. Combined ET and LLF (EL), a little formula designed following the TCM theories and clinical experience, has been used to treat asthmatic patients in China for three decades [11]. Preclinical studies reported that ET treatment was able to decrease airway inflammation and airway hyperresponsiveness, adjust the disequilibrium of Th1/Th2, and protect HPA axis [12].

According to TCM theories, the use of GCs in clinical practice is able to injure kidney function and leads to kidney deficiency [32]. And the suppression of the HPA axis and the decrease of GR content or GR binding caused by administration of GC were proved to play key roles in pathogenesis of kidney deficiency syndrome [33, 34]. So many TCM doctors take the methods of tonifying kidney to prevent and treat the side effects of GC [35, 36]. In our previous researches, we demonstrated that the decoction of EL in combination with dexamethasone (Dex) had a better anti-inflammatory effect, increased the mRNA and protein expression of GR in lung, and prevented the inhibition of HPA axis and loss of bone mass in the asthmatic rats, compared with only using Dex [13, 14]. It is induced that EL could increase the sensitivity and decrease the side effects of GC. However, the underlying molecular mechanism of EL combined with inhaled GCs is not yet well understood.

Alterations in the GR expression are one of the potential mechanisms that would explain GC insensitivity. Abnormalities in GR binding to GCs have been described in the lymphocytes of patients with GC-resistant asthma or in asthma in general [37]. GR expression is downregulated by GC administration in bronchial epithelial cells, and GR binding activity and GC responsiveness are depressed by proinflammatory stimuli in asthma [38, 39]. Although GCs therapy could suppresses allergen-induced airway inflammation, epithelial cell shedding is not corrected and asthma cannot be cured completely [40, 41]. Reduction of GR mRNA and protein expression in the lungs of mice exposed to allergen has been reported [42]. These findings of decreased expression of GR in asthma patients or allergic mice suggest that an overall reduction in GR may compromise asthma immune response [43]. According to the results, it could be observed that the levels of GR binding capability in BALF lymphocytes and GR mRNA expression in lung tissues were markedly decreased in the OVA-induced asthmatic rats, suggesting impaired GR function in asthma. Treatment with EL and coadministration of Bun and EL could increase GR binding capability and GR mRNA expression in airway. This increase might improve GC sensitivity when used for the treatment of asthma.

GR gene expresses 2 splicing isoforms, that is, GR*α* and GR*β* [44]. GR*β* with a truncated ligand binding domain does not bind GCs and was found to be a dominant-negative inhibitor of the classic GR*α* [45]. Increased GR*β* expression has been reported in bronchial asthma [46]. Certain proinflammatory cytokines upregulate GR*β* expression. And an association between increased GR*β* expression and GC insensitivity has been confirmed in asthma [26]. There is growing evidence that high expression of GR*β* could be responsible for the development of insensitivity to GCs in a variety of inflammatory disorders [47]. It has been proposed that GC resistance could result from the imbalance between GR*α* and GR*β*, though little is known whether the GR*α*/GR*β* ratio could determine the level of GC responsiveness [48]. Additionally, it has been shown that GR*β* did not interfere with GR-mediated transrepression and GR*α* excess seems to overcome its inhibitory action [49]. As shown in this study, the mRNA expression of GR*β* increased, while the mRNA and protein expressions of GR*α* were downregulated in the OVA-induced asthmatic rats and those treated with Bun, which was in accord with the results of GR binding capability and mRNA expression in airway. Both administration of EL and coadministration of Bun and EL could increase GR*α* protein and mRNA expression, decrease GR*β* mRNA expression, and correct GR*α*/GR*β* ratio in the protein or mRNA level. These findings indicate that EL could promote the pharmacological actions of GR*α*-mediated GC and inhibit GR*β*-mediated insensitivity of GC, suggesting that the coadministration of Bun and ET is beneficial to improve anti-inflammatory effects of GCs and prevent GC resistance.

The ligand binding activity of GR is related to the changes of HSP90. The inactive form of GR in the cytoplasm is bound to HSP90. This interaction facilitates binding of GC to GR and depends on the relative amounts of the interacting components, GR and HSP90 [50]. HSP90 content is maybe related to the GR sensitivity to GCs. HSP90 does not inhibit GR function solely by steric interference; rather, HSP90 seems to facilitate the subsequent response of apoGR to the hormonal signal (Vamvakopoulos, 1993). In this report, we found that the protein and mRNA expressions of HSP90 in airway were significantly decreased after modeling; besides, there were decreased GR*α*/HSP90 ratio and increased GR*β*/HSP90 ratio in the protein or mRNA level. Both administration of EL and coadministration of Bun and EL could increase HSP90 expression and modulate the ratio of GR*α* to HSP90 or GR*β* to HSP90. We could infer from our results that the regulation mechanisms of EL on GC sensitivity may be associated with HSP90 expression and the balance between GR and HSP90.

## 5. Conclusions

In this study, we have found that EL combined with Bun had superior effects in increasing BALF lymphocyte apoptosis and GR binding capacity, upregulating the protein and mRNA expressions of GR, GR*α*, and HSP90, improving GR*α*/GR*β* ratio and GR*α*/HSP90 ratio in protein or mRNA level, and inhibiting GR*β* expression and GR*β*/HSP90 ratio in the mRNA level. Moreover, there was a significant correlation between GR*α* and GR*β* in the protein level or between GR*α* and HSP90 both in the protein and in mRNA levels. Our results demonstrated that EL could effectively raise the sensitivity of Bun via balancing GR/HSP90. These findings will be beneficial for the treatment of asthma in the future.

## Figures and Tables

**Table 1:** Primers used for qPCR analysis.

Primer	Forward primer	Reverse primer
GR	GCCCTGGGTTGGAGATCATAC	CATGCAGGGTAGAGACATTCTC
GR*α*	GCGACAGAAGCAGTTGAGTCATC	CCATGCCTCCACGTAACTGTTAG
GR*β*	GCGCTTGAGGCTAAGATAGCT	CCCATGTTTCTGCCTCTTTCTTTG
HSP90	CCTGGGAAGCCCCCG	TTGTAGACATGAGCAGAGAGCC
18S	CCGGTACAGTGAAACTGCGA	GATAAATGCACGCGTTCCCC

## Abbreviations

BALF: Bronchoalveolar lavage fluid
Bun: Budesonide
FCM: Flow cytometry
FITC: Fluorescein isothiocyanate
GC: Glucocorticoid
GR: Glucocorticoid receptor
EF: Epimedii Folium
EL: Epimedii Folium and Ligustri Lucidi Fructus
LLF: Ligustri Lucidi Fructus
HPA: Hypothalamic-pituitary-adrenal
HSP90: Heat shock protein 90
IHC: Immunohistochemistry
ICC: Immunocytochemistry
IOD: Integral optical density
i.p.: Intraperitoneal injection
MFI: Mean fluorescence intensity
OVA: Ovalbumin
PE: Phycoerythrin
qPCR: quantitative real-time PCR
SD: Standard deviation
TUNEL: TdT-dUTP terminal nick-end labeling
WB: Western blotting.
